# Personal GHG emissions accounting and the driving forces decomposition in the past 10 years

**DOI:** 10.1007/s43979-023-00045-9

**Published:** 2023-02-08

**Authors:** Yuxiao Zhou, Jiyang Li, Jicui Cui, Hui Wang, Chuan Wang, Ruina Zhang, Ying Zhu, Nanwen Zhu, Ziyang Lou

**Affiliations:** 1 Shanghai Engineering Research Center of Solid Waste Treatment and Resource Recovery, School of Environmental Science and Engineering, Shanghai Jiao Tong University, 800 Dongchuan Road, Minhang District, Shanghai, 200240 China; 2 China-UK Low Carbon College, Shanghai Jiao Tong University, Shanghai, 201306 China; 3 Shanghai Environmental Sanitary Engineering Design Institute Co., Ltd, Shanghai, 200232 China; 4 Advanced Materials Institute, Qilu University of Technology (Shandong Academy of Sciences), Jinan, 250014 China; 5 China Institute for Urban Governance, Shanghai Jiao Tong University, Shanghai, 200240 China; 6 Shanghai Jiao Tong University Sichuan Research Institute, Shanghai, China

**Keywords:** Personal GHG emissions, Consumer lifestyle approach, Waste disposal, LMDI, Driving forces

## Abstract

Personal greenhouse gas (P_GHG_) emissions were crucial for achieving carbon peak and neutrality targets. The accounting methodology and driving forces identification of P_GHG_ emissions were helpful for the quantification and the reduction of the P_GHG_ emissions. In this study, the methodology of P_GHG_ emissions was developed from resource obtaining to waste disposal, and the variations of Shanghainese P_GHG_ emissions from 2010 to 2020 were evaluated, with the driving forces analysis based on Logarithmic Mean Divisia Index (LMDI) model. It showed that the emissions decreased from 3796.05 (2010) to 3046.87 kg carbon dioxides (CO_2_) (2014) and then increased to 3411.35 kg CO_2_ (2018). The emissions from consumptions accounted for around 62.1% of the total emissions, and that from waste disposal were around 3.1%, which were neglected in most previous studies. The P_GHG_ emissions decreased by around 0.53 kg CO_2_ (2019) and 405.86 kg CO_2_ (2020) compared to 2018 and 2019, respectively, which were mainly affected by the waste forced source separation policy and the COVID-19 pandemic. The income level and consumption GHG intensity were two key factors influencing the contractively of GHG emissions from consumption, with the contributing rate of 169.3% and − 188.1%, respectively. Energy consumption was the main factor contributing to the growth of the direct GHG emissions (296.4%), and the energy GHG emission factor was the main factor in suppressing it (− 92.2%). Green consumption, low carbon lifestyles, green levy programs, and energy structure optimization were suggested to reduce the P_GHG_ emissions.

**Supplementary Information:**

The online version contains supplementary material available at 10.1007/s43979-023-00045-9.

## Highlights

● The boundary of P_GHG_ emissions developed from resource obtaining to waste disposal.

● Shanghainese P_GHG_ emissions varied from 3004.64 to 3796.05 kgCO_2_ in 2010-2020.

● Around 405.86 kg CO_2_ decreased with the reduced consumption by COVID-19 lockdown.

● Energy consumption was the main contributor to DP_GHG_ emissions (296.4%) increase.

● The GHG intensity was the main contributor to IP_GHG_ emissions reduction (− 188.1%).

**Supplementary Information:**

The online version contains supplementary material available at 10.1007/s43979-023-00045-9.

## Introduction

The extreme weather resulting from greenhouse gas (GHG) emissions has been confirmed to be a severe problem to the nature and social system [25]. GHG emissions from personal behaviors contributed significantly to the total amounts through the direct energy use and the embodied parts in the daily consumptions [20]. It was estimated that more than 70% of the total GHG emissions were attributed to personal behaviors in developed countries [3]. To reach the target of achieving the carbon peak and neutrality target and limiting the global warming to 1.5 °C above preindustrial levels, the efforts from the public were crucial [34], where a premise of that was the well-established methodology that guiding the concise accounting of the personal GHG (P_GHG_) emissions, and a case study could reflect the variation trends and identify the critical emission source and the driving forces.

Recent studies have shown increased attention to GHG emissions at the personal and household level [9, 42]. The quantifications of the personal and household GHG emissions have been studied in India [1], China [10, 19, 27], USA [5], Japan [22] and Europe [7, 18] from the regional to national scales. It was commonly acknowledged that personal behavior played a fundamental role in implementing of GHG reduction policies in these studies. However, when considering the boundary of the P_GHG_ emissions, the scopes varied largely. Some studies focused on the indirect emissions from household consumptions, including the process of producing, processing and obtaining of goods and services [37, 39]. Some studied the personal energy consumption and corresponding direct GHG emissions when products using [1, 24]. And some other studies considered both emissions [11, 38]. While few studies have put forward the complete methodologies, which extended from the resource obtaining to waste disposal for P_GHG_ emissions.

China, as the largest developing country in the world, contributed to the highest GHG emissions, with the amounts of 11.9 billion tons in 2021, accounting for around 33% of the total emissions [14]. To achieve the sustainable development goals (SDGs) on climate change (Target 13) [33], China has committed a series of reduction targets, including reaching peak GHG emissions by 2030 and becoming carbon neutral by 2060. To reduce the GHG emissions, the carbon trading system has been implemented for the energy and industry sectors in China that the businesses needed to pay for the parts above the limitations [17]. While the P_GHG_ emissions, as the important source, have not been regulated effectively. Many cities in China, such as Guangdong, Shandong, Zhejiang, Shanghai, are exploring carbon inclusion mechanism to reduce personal GHG emissions. It should be noted that the accurate accounting of P_GHG_ emissions should be the basis for implementing the carbon inclusion campaign [43]. While the complete and reliable methodology has not been put forward.

As the above illustrated, the accurate accounting of P_GHG_ emissions, which extended from resource obtaining to waste disposal was crucial for realizing the carbon peak and neutrality targets. Besides, the socio-economic factors that influenced the P_GHG_ emissions were urgent to be recognized, which would help policy making to reduce the GHG emissions from the public perspective. The Shanghainese average P_GHG_ emissions were estimated as a case study, where was one of the most developed cities in China and could be a reference for the other developing countries and cities. Moreover, Shanghai have undergone the deep transformation of waste forced source separation policy and COVID-19 pandemic, which could have large influences on the P_GHG_ emissions. Identifying that could help us understand the impact of major policies or emergencies on P_GHG_ emissions.

The main objectives of this study included: (1) The boundary of P_GHG_ emissions and accounting methodology were proposed; (2) The variations and distributions of the P_GHG_ emissions from the direct energy use and indirect consumption and final waste disposal were quantified; (3) The influences of the forced source separation policy and COVID-19 pandemic on living behaviors and the corresponding P_GHG_ emissions were identified; (4) The driving forces of the P_GHG_ emissions were analyzed.

The remainder of this article was organized as follows. Section 2 presented the analytical methods and data sources. Section 3 firstly presented the variations of Shanghainese P_GHG_ emissions in the steady development period from 2010 to 2018, and then studied the influence of forced source separation policy and COVID-19 pandemic on that in 2019 and 2020. In Section 4, the driving forces of the P_GHG_ emissions were analyzed, and the policy implications related to P_GHG_ emissions were proposed. Section 5 proposed the conclusions.

## Materials and methods

### P_GHG_ emissions accounting methodology

**Fig. 1:**
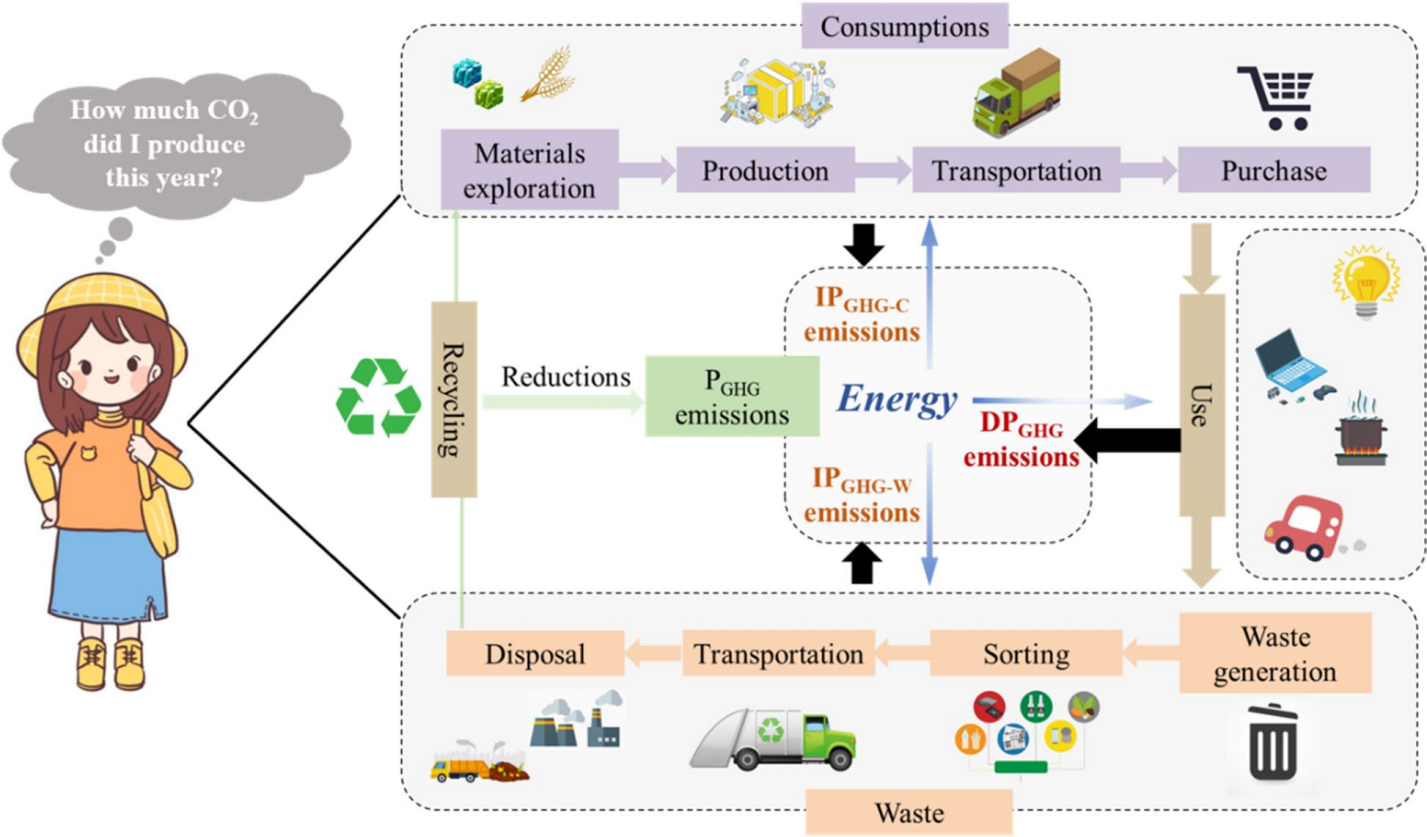
System boundary and the detailed scope of the P_GHG_ emissions

P_GHG_ emissions came both from the direct emissions from energy use (i.e., direct emissions) and the embodied emissions from their expenditure on goods and services as well as the final waste disposal during the entire life cycle (i.e., indirect emissions) [35]. The boundary and the detailed scope of the P_GHG_ emissions is shown in Fig. 1. The P_GHG_ emissions in year t (P_GHG_^t^) could be obtained by the following equation:

1 $${{\textrm{P}}_{\textrm{GHG}}}^{\textrm{t}}={{\textrm{DP}}_{GHG}}^{\textrm{t}}+{{\textrm{IP}}_{GHG}}^{\textrm{t}}$$

Where DP_GHG_^t^ and IP_GHG_^t^ are direct and indirect personal GHG emissions in year t, respectively.

### Estimation of DP_GHG_ emissions

The DP_GHG_ emissions were mainly generated from residential energy use, such as lighting, appliances, cooking, space heating, water heating, and private travel. Seven types of energy consumed were considered, including coal, natural gas, liquefied petroleum gas (LPG), electricity, coal gas, gasoline and diesel, according to Shanghai Statistical Yearbook [28]. The study applied the emissions coefficient method (ECM) followed by the Intergovernmental Panel on Climate Change (IPCC) guideline [15] to calculate the DP_GHG_^t^:

2 $${{\textrm{DP}}_{GHG}}^{\textrm{t}}=\sum\nolimits_j{E}_j^t\times {EF}_j$$

where *DP*_*GHG*_^*t*^ represented the GHG emissions from fossil fuel in year *t*, with unit of kg carbon dioxides (CO_2_); *E*_*j*_^*t*^ is the energy type *j* consumption in year *t*, with units of kg (various gases are measured by m^3^); *EF*_*j*_ is the emission factor of fuel type *j*, with units of kg CO_2_/kg (or kg CO_2_/m^3^). The emission factor for the electricity was based on the energy mix in China with the dynamic changes [30] since the emission factor was decreasing with more proportion of the clean energy. The detailed emission factors can be seen in the supplementary information (SI).

### Estimation of IP_GHG_ emissions

#### Consumptions

The indirect emissions that occurred in the production of daily consumptions were assessed by the consumer lifestyle approach (CLA), which was put forward by Bin and Dowlatabadi [5] and often used in the evaluation of residential indirect GHG emissions [8, 37, 38]. The Shanghainese average consumption expenditure values were taken to calculate indirect energy use by personal behaviors. The consumer expenditures include food, clothing, residence, household facilities and services, education cultural, and recreation services, medicines and health care, communication services and residence. The full list of consumer behavior to cause indirect GHG emissions is shown in Table 1. The indirect personal GHG emissions from consumptions (IP_GHG-C_ emissions) could be calculated as follows:

3 $${{\textrm{IP}}_{\textrm{GHG}-\textrm{C}}}^t=\sum\nolimits_i\left({CI}_i^t\times {X}_i^t\right)$$

Where *IP*_*GHG-C*_^*t*^ was the indirect GHG emissions from personal consumption in year t, with unit of kg CO_2_; *CI*_*i*_^*t*^ refereed to the consumption GHG intensity of the sector i in year t, with unit of kg CO_2_/ CNY (Chinese Yuan) (*CI*_*i*_^*t*^ *= C*_*i*_^*t*^*/ G*_*i*_^*t*^, *C*_*i*_^*t*^ referred to the sum of CO_2_ emission of the sector i in year t, while *G*_*i*_^*t*^ referred to the sum of the added value of the sector i in year t); *X*_*i*_^*t*^ refers to the per capita expenditure of the sector *i* in year t, with unit of CNY.

**Table 1:** Sectors related to household consumer behaviors

Consumer expenditure	Related sectors
Food	Processing of food from agricultural products; Manufacture of foods; Manufacture of liquor, beverages and refined tea; Manufacture of tobacco.
Clothing	Manufacture of textile; Manufacture of textile, wearing apparel and accessories; Manufacture of leather, fur, feather and related products and footwear.
Household facilities and services	Processing of timber, manufacture of wood, bamboo, rattan; Manufacture of furniture.
Education, cultural and recreation services	Manufacture of paper and paper products; Printing and reproduction of recording media; Manufacture of articles for culture, education, arts and crafts.
Medicine and medical services	Medical and pharmaceutical products.
Communication services	Electronic and telecommunications equipment.
Residence	Production and supply of gas and heat; Production and supply of water.

It was assumed that the GHG emissions could be linked to the energy efficiency in industries and monetary flows. The domestic energy efficiency and the related GHG intensity was applied, while it should be noted that a part of the consumption expenditure was the foreign imported products, and the GHG intensity differed due to the difference in the energy structure and technical level [12]. As the top five imported countries for China were the United States, Japan, Korea, Germany, and Austria in 2021, all of which had higher energy efficiencies than China, and it was assumed that the GHG intensity could be the upper limit [11].

#### Waste generated features

Residents did not need to pay for waste disposal in China, and the GHG emissions generated in the waste disposal were not included in the CLA analysis. However, from the perspective of the whole life cycle, the GHG emissions of waste disposal should be included in the P_GHG_ emissions, which were often ignored in many studies [35, 37]. The indirect personal GHG emissions from waste disposal (IP_GHG-W_) could be calculated as follows:

4 $${IP_{GHG-W}}^t={\sum}_j\left[{WT}_j^t\times {\sum}_i\left({DP}_i^t\times {EF}_i\right)\right]$$

Where *IP*_*GHG-W*_^*t*^ was the indirect personal GHG emissions of waste disposal in year *t*, with unit of kg CO_2_; *WT*_*j*_^*t*^ referred to the amount of the waste *j* in year *t* (such as mixed waste, wet waste, recyclables), with unit of ton; *DP*_*i*_^*t*^ referred to the percentage of the technology *i* in year *t* (such as incineration, landfilling, composting, and anaerobic digestion); *EF*_*i*_ referred to the emission factor of the technology *i*, with unit of kg CO_2_/ton. The emission factors were assigned according to our previous works and the literature results [6, 21, 23, 40, 41, 44], which is shown in SI.

### Driving forces analysis and logarithmic mean Divisia index (LMDI) model

The LMDI model was a useful technique that could decompose the various indexes and quantify the contributions of different factors to the growth of burdened issues and rationally allocate reduction targets. Numerous studies had applied the LMDI model in GHG emissions analysis [36]. According to the estimation results, the DP_GHG_ emissions and IP_GHG_ emissions could be decomposed as eqs. (5) and (6), respectively.

5 $${\displaystyle \begin{array}{c}{DP_{GHG}}^t=\sum\limits_{j=1}^7\frac{{{DP_{GHG}}^t}_j}{E_{j,t}}\times \frac{{E^t}_j}{EX^t}\times \frac{EX^t}{W^t}\times {W}^t\\ {}=\sum\nolimits_{j=1}^7{CE^t}_j\times {GI^t}_j\times {TS}^t\times {W}^t\end{array}}$$

Where *DP*_*GHG*_^*t*^_*j*_ was the personal direct GHG emission emissions in the year *t* (kg CO_2_, j = 1 ~ 7, which represented coal, natural gas, LPG, electricity, coal gas, gasoline, and diesel). *E*^*t*^_*j*_ represented the consumption amount of energy *j* per capita in year t (kg of standard coal equivalent, kg ce). *EX*^*t*^ represented the total energy usage amount in year t. *W*^*t*^ represented the consumption expenditure on energy *j* per capita (CNY). Then four driving forces can be defined as follows. *CE*^*t*^_*j*_ was the amount of GHG emission per unit of energy consumption, which could reflect the GHG emission factor effect; *GI*_*,*_^*t*^_*j*_ represented the proportion of different energy types in total energy consumption, which could represent the energy structure effect. *TS*^*t*^_*j*_ represented the energy amount per unit expenditure, which could reflect the energy price effect. *W*^*t*^_*j*_ represented energy consumption amount, and was defined as energy consumption effect.

6 $${\displaystyle \begin{array}{c}{IP_{GHG}}^t=\sum\limits_{i=1}^7\frac{{{IP_{GHG}}^t}_i}{{W^t}_i}\times \frac{{W^t}_i}{EW^t}\times \frac{EW^t}{G^t}\times {G}^t\\ {}=\sum\nolimits_{i=1}^7{ED^t}_i\times {NU^t}_i\times {WT^t}_i\times {GT^t}_i\end{array}}$$

Where *IP*_*GHG*_^*t*^_*i*_ was the personal indirect GHG emission emissions in the year t (kg CO_2_). *W*^*t*^_*i,*_ represented the consumption expenditure of sector *i* per capita in year *t* (CNY). *EW*^*t*^ represented the total consumption expenditure per capita in year *t*. *G*^*t*^ represented the GDP per capita in year t (CNY). Then four driving forces can be defined as following. *ED*^*t*^_*i,*_ was the amount of GHG emission per unit of consumption expenditure of sector *i*, which could reflect the emission intensity effect; *NU*^*t*^_*i,*_ was the proportion of different sectors of total consumption expenditure, which could present the consumption structure effect. *WT*^*t*^_*i*_ was the ratio of consumption to income, which could reflect the consumption willing effect. *GT*^*t*^_*i,*_ was defined as the income level effect.

The accumulative effects of the driving forces of DP_GHG_ and IP_GHG_ could be calculated by Eqs. (7)-(16).

7 $$\Delta {DP}_{GHG}=\Delta {DP_{GHG}}^t-\Delta {DP_{GHG}}^{t-1}=\Delta CE+\Delta GI+\Delta TS+\Delta W$$

8 $$\Delta CE=\sum\nolimits_{j=1}^7\frac{{{DP_{GHG}}^t}_j-{{DP_{GHG}}^{t-1}}_j}{\ln {{DP_{GHG}}^t}_j-\ln {{DP_{GHG}}^{t-1}}_j}\times \ln \frac{{CE^t}_j}{{CE^{t-1}}_j}$$

9 $$\Delta GI=\sum\nolimits_{j=1}^7\frac{{{DP_{GHG}}^t}_j-{{DP_{GHG}}^{t-1}}_j}{\ln {{DP_{GHG}}^t}_j-\ln {{DP_{GHG}}^{t-1}}_j}\times \ln \frac{{GI^t}_j}{{GI^{t-1}}_j}$$

10 $$\Delta TS=\sum\nolimits_{j=1}^7\frac{{{DP_{GHG}}^t}_j-{{DP_{GHG}}^{t-1}}_j}{\ln {{DP_{GHG}}^t}_j-\ln {{DP_{GHG}}^{t-1}}_j}\times \ln \frac{TS^t}{TS^{t-1}}$$

11 $$\Delta W=\sum\nolimits_{j=1}^7\frac{{{DP_{GHG}}^t}_j-{{DP_{GHG}}^{t-1}}_j}{\ln {{DP_{GHG}}^t}_j-\ln {{DP_{GHG}}^{t-1}}_j}\times \ln \frac{W^t}{W^{t-1}}$$

12 $$\Delta {IP}_{GHG}=\Delta {IP_{GHG}}^t-\Delta {IP_{GHG}}^{t-1}=\Delta ED+\Delta NU+\Delta WT+\Delta GT$$

13 $$\Delta ED=\sum\nolimits_{i=1}^7\frac{{{IP_{GHG}}^t}_i-{{IP_{GHG}}^{t-1}}_i}{\ln {{IP_{GHG}}^t}_i-\ln {{IP_{GHG}}^{t-1}}_i}\times \ln \frac{{ED^t}_i}{{ED^{t-1}}_i}$$

14 $$\Delta NU=\sum\nolimits_{i=1}^7\frac{{{IP_{GHG}}^t}_i-{{IP_{GHG}}^{t-1}}_i}{\ln {{IP_{GHG}}^t}_i-\ln {{IP_{GHG}}^{t-1}}_i}\times \ln \frac{{NU^t}_i}{{NU^{t-1}}_i}$$

15 $$\Delta WT=\sum\nolimits_{i=1}^7\frac{{{IP_{GHG}}^t}_i-{{IP_{GHG}}^{t-1}}_i}{\ln {{IP_{GHG}}^t}_i-\ln {{IP_{GHG}}^{t-1}}_i}\times \ln \frac{{WT^t}_i}{{WT^{t-1}}_i}$$

16 $$\Delta GT=\sum\nolimits_{i=1}^7\frac{{{IP_{GHG}}^t}_i-{{IP_{GHG}}^{t-1}}_i}{\ln {{IP_{GHG}}^t}_i-\ln {{IP_{GHG}}^{t-1}}_i}\times \ln \frac{{GT^t}_i}{{GT^{t-1}}_i}$$

### Data source

The household energy consumption per capita was taken from Shanghai Statistical Yearbook [28]. The consumption expenditure of Shanghai’s residents was taken from Shanghai Statistical Yearbook [28]. The gross outputs of different sectors were taken from China Industrial Statistical Yearbook [30] and China Statistical Yearbook [31]. The energy consumptions of different sectors were taken from China Energy Statistical Yearbook [32]. The data of the MSW generation and disposal amounts in Shanghai were retrieved from the statistical yearbook published by Shanghai Landscaping and City Appearance Administrative Bureau [29]. The global warming potential (GWP) of CH_4_ and N_2_O were 28 and 273 times of CO_2_ separately according to IPCC AR6.

## Results

### The variation and distribution of Shanghainese average P_GHG_ emissions in the steady development period from 2010 to 2018

**Fig. 2:**
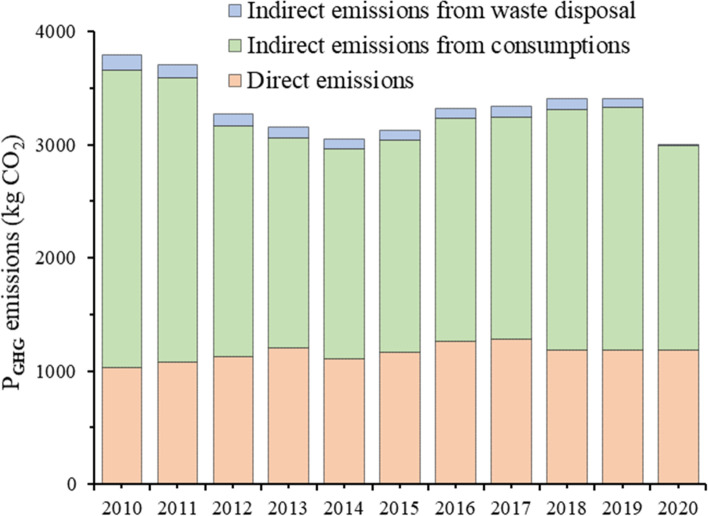
The variations of Shanghainese average P_GHG_ emissions from 2010 to 2020

The variations of Shanghainese average P_GHG_ emissions from 2010 to 2020 can be seen in Fig. 2. Focusing on the steady development period from 2010 to 2018. It decreased from 3796.05 kg CO_2_ per capita to 3046.87 kg CO_2_ per capita from 2010 to 2014 and then increased to 3411.35 kg CO_2_ per capita in 2018. Specifically, the DP_GHG_ emissions took around 34.8%, and the IP_GHG_ emissions took around 65.2% of the total emissions in average.

#### Direct GHG emissions from energy use

**Fig. 3:**
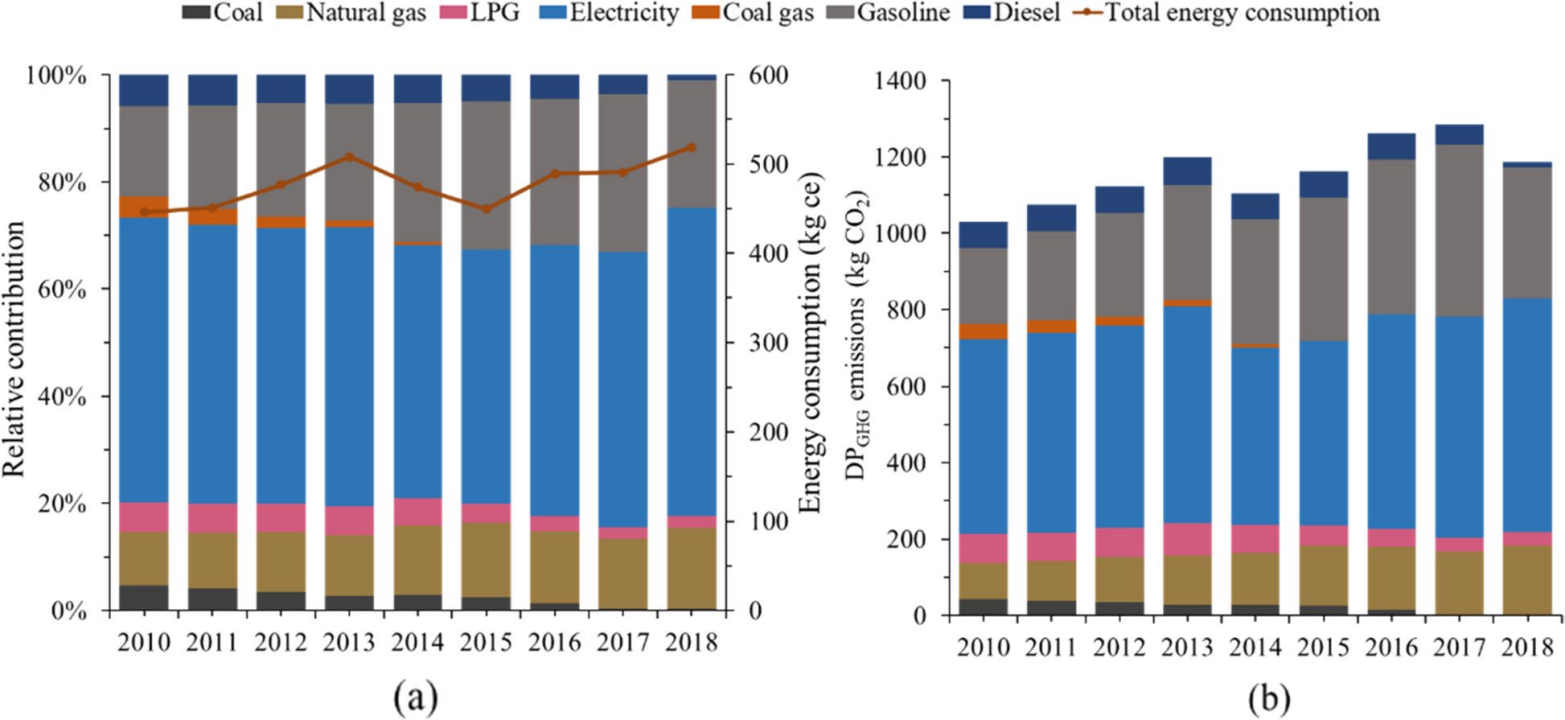
**a** The Shanghainese average energy consumption and the proportion of different categories (2010-2018); **b** The variation of Shanghainese average DP_GHG_ emissions (2010-2018)

The Shanghainese average energy consumption and the corresponding DP_GHG_ emissions from 2010 to 2018 are presented in Fig. 3, which generally showed a rising trend, increasing from 1030.01 to 1187.49 kg CO_2_, with the growth of around 15.29%. The emissions from electricity, natural gas and gasoline were the top three emissions sources. It could be seen that the variations of DP_GHG_ emissions were generally related to the energy use, while not completely consistent. It may be explained by the lower GHG emissions intensity of energy consumption, which turned from 2.31 to 2.29 kg CO_2_/ kg ce contributed by the changes in energy structure and electricity mix.

It was noticed that there was a sharp rise of energy consumption in 2013 and then decreased rapidly in 2014, which was mainly attributed to the abnormal climate. There were 47 high-temperature days in the summer of 2013 in Shanghai, much higher than that in 2012 (24 days) and 2014 (8 days), and the electricity demand for air conditioners rose sharply, which also reflected the significant impact of global climate changes on our lives [13]. It could also be found that the GHG emissions that related with the personal transportation in Shanghai have changed significantly. The consumption of gasoline increased from 41.8 kg in 2010 to 116.8 kg per capita in 2017, which was mainly attributed to the acceleration of urbanization. While affected by Shanghai’s road restriction policy and the promotion of new energy vehicles, the demand in gasoline reduced to 89.00 kg per capita in 2018.

#### Indirect GHG emissions from consumption and waste disposal

**Fig. 4:**
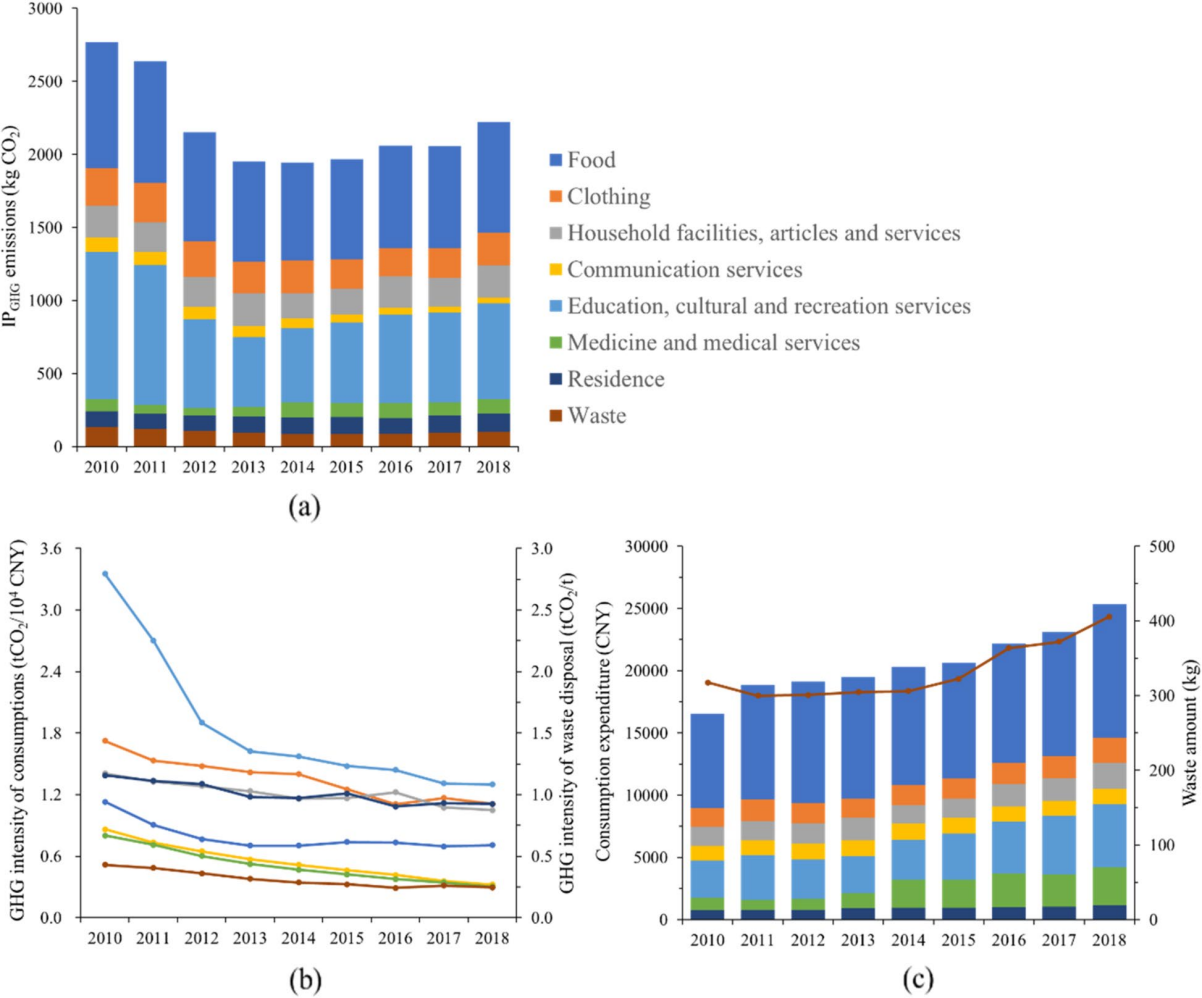
**a** The variations of Shanghainese average IP_GHG_ emissions (2010-2018); **b**: The GHG intensity of different sectors (2010-2018); **c** The consumption expenditure and waste amount per capita in Shanghai (2010-2018)

The variations of Shanghainese IP_GHG_ are shown in Fig. 4 (a), which presented as a “U-shaped” trend from 2010 to 2018. The maximum emission was observed in 2010, with the value of 2766.05 kg CO_2_ per capita. The emissions kept decreasing from 2010 to 2014, reaching the minimum value in 2014 (1942.59 kg CO_2_). And then, it turned to increase until 2018, with the value of around 2223.85 kg CO_2_.

The GHG intensity of the consumptions and the waste disposal are shown in Fig. 4 (b). It could be found that the consumption GHG intensity showed a falling trend, and the falling rate was relatively fast before 2014, and then it slowed down. The falling trend was mainly attributed to the improved energy efficiency by the technological innovation [37]. It was also observed that the GHG intensity of the waste disposal decreased from 0.43 to 0.25 t CO_2_/t waste, which was mainly attributed to the increased proportion of incineration instead of landfilling in the waste disposal structure in Shanghai. With the stable development of the Chinese economy, the Shanghainese consumption expenditure and the waste amount also kept increasing from 2010 to 2018.

The results showed that the expenditures for food, education, cultural, and recreation services contributed to the most GHG emissions of the IP_GHG_ in Shanghai from 2010 to 2018, while their driving factors differed. Combining the GHG intensity and consumption expenditure, it could be found that GHG emissions from food were mainly driven by the highest expenditure, and the education, cultural and recreation services were driven by the higher GHG intensity. Besides, it could be found that the main reason for the reduction of IP_GHG-C_ from 2010 to 2014 was the decrease in GHG intensity. For example, the GHG intensity of education, cultural and recreation services decreased by 53.1%, and food decreased by 38.2%. Although the personal expenditure increased, the IP_GHG_ showed a relatively large decline. From 2014 to 2018, the decrease rate of GHG intensity was slowed down, while the consumption expenditure increased by around 24.8%, which overly contributed around 14.7% to the increase of IP_GHG_.

### The variation of Shanghainese average P_GHG_ emissions in 2019 and 2020 when affected by forced source separation policy and COVID-19 pandemic

The Shanghainese average P_GHG_ emissions, which continued to increase from 2014 to 2018, seemed to show inflection points in 2019 and 2020 in Fig. 2, which was mainly affected by the waste forced source separation policy and the COVID-19 pandemic. The P_GHG_ emissions decreased by around 0.53 kg CO_2_ from 2018 to 2019, which was mainly due to the negative GHG emissions of waste source recycling. The P_GHG_ emissions decreased by 405.86 kg CO_2_ from 2019 to 2020, which was mainly attributed to the decreased IP_GHG_ emissions by less consumption willingness.

#### The influence of forced source separation policy

Affected by the forced source separation policy, the Shanghainese average GHG emissions from waste disposal decreased from 100.27 kg CO_2_ (2018) to 77.02 kg CO_2_ (2019) and finally reached 11.15 kg CO_2_ (2020).

**Fig. 5:**
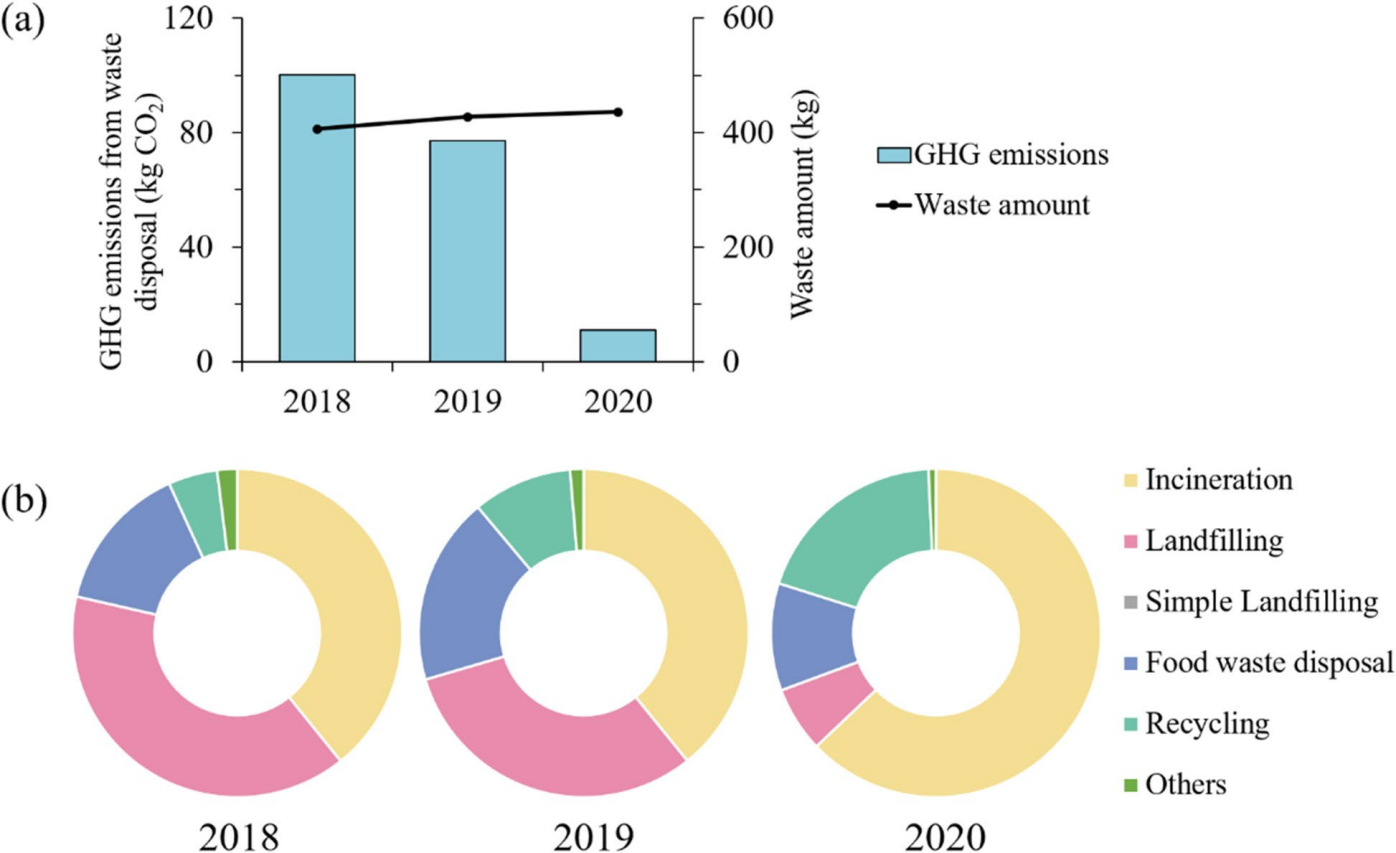
**a** The variations of Shanghainese average GHG emissions from waste disposal before and after forced source separation; **b** The waste disposal structure before and after forced source separation

It could be seen in Fig. 5 that the GHG emissions were not consistent with the waste generation amount, which was mainly attributed to the changing waste disposal structure. The proportion of landfilling, which had the largest GHG intensity, has decreased from 39.4% (2018) to 31.2% (2019), and finally reached 6.5% (2020). Meanwhile, the separated food waste increased by around 33.2% in 2019 due to the more proportion of the food waste disposal technologies, such as anaerobic digestion and composting, which had lower GHG intensity. While with the influence of the control measures for COVID-19 pandemic, the proportion of food waste disposal was reduced in 2020. Another notable influence was the change in the recyclables, which increased by around 113.7% and 107.5% in 2019 and 2020, respectively. Recycling could reduce GHG emissions by substituting the production of upstream substances, making recycling a GHG savings behavior. The GHG emissions of recycling decreased from − 8.38 kg CO_2_ (2018) to − 36.18 kg CO_2_ (2020), and it could be forecasted that with the deep promotion of source separation, waste disposal could even become a reduction sector of P_GHG_ emissions.

#### The influence of COVID-19 pandemic on P_GHG_ emissions

**Fig. 6:**
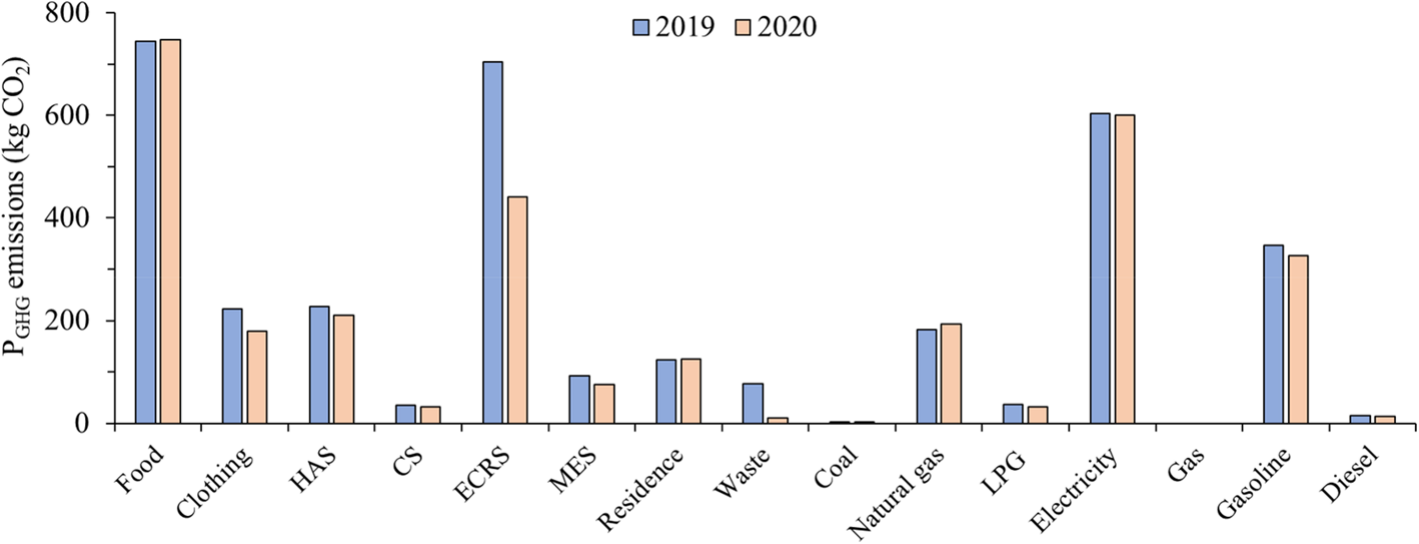
The comparison of Shanghainese average P_GHG_ emissions before and after COVID-19 Pandemic. (HAS: Household facilities, articles and services; CS: Communication services; ECRS: Education, cultural and recreation services; MES: Medicine and medical services)

During the COVID-19 lockdown, many human activities stopped as a result of curfew, and hence contributing to the change in the GHG emissions, especially on the IP_GHG_ emissions. The decline of around 405.86 kg CO_2_ was found in 2020 compared to 2019. The detailed changes of P_GHG_ emissions were shown in Fig. 6.

For IP_GHG_ emissions, the results showed that the emissions from education, cultural and recreation services decreased by around 37.3%, which was the largest contributor to the decline in IP_GHG_ emissions. The change was mainly attributed to the strict safety measures on the education and entertainment venues. The second largest decline of around 19.4% was on the clothing, which was mainly attributed to the reduced demand for the clothing due to the lockdown measures. For DP_GHG_ emissions, the demand for gasoline reduced by around 6.0%, this is mainly because non-essential travel was not allowed during the COVID-19 lockdown.

## Discussion

### Driving factors analysis of P_GHG_ emissions

**Fig. 7:**
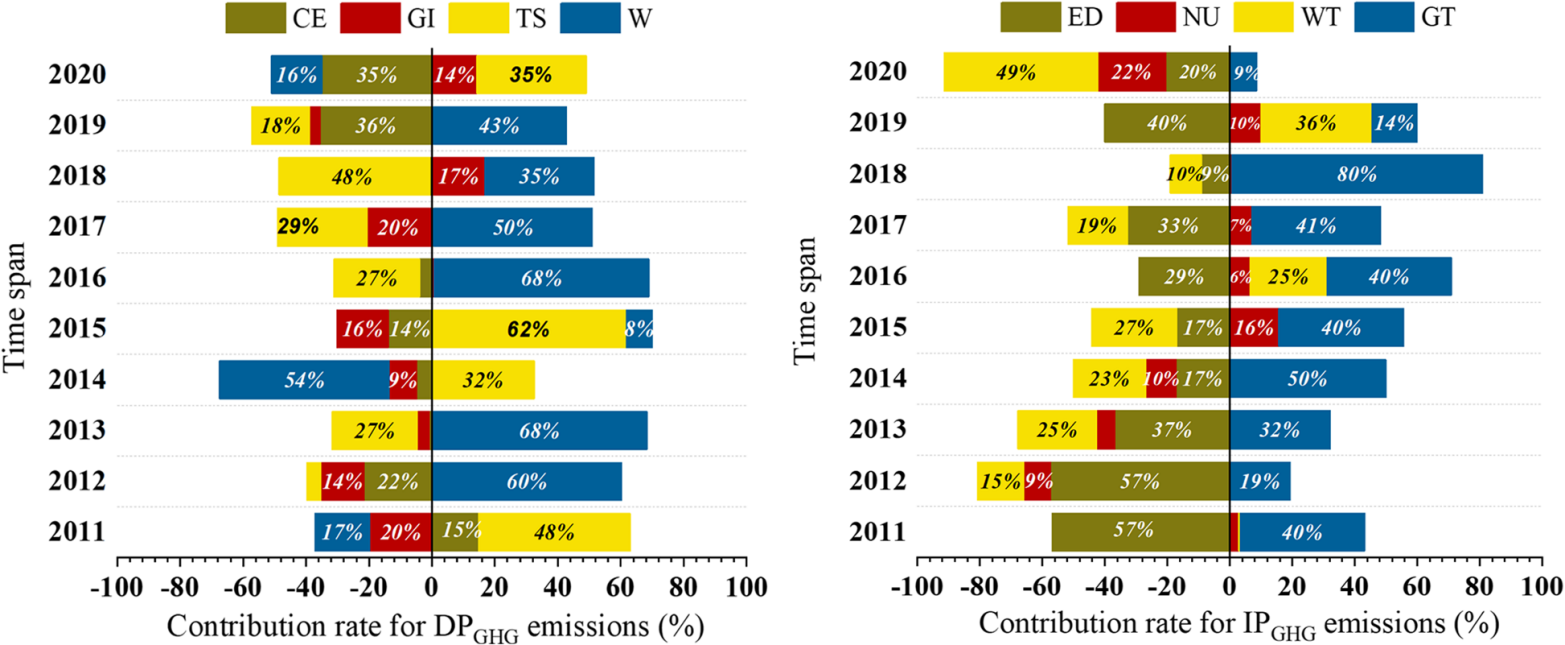
Driving forces of Shanghainese personal GHG emissions during 2011–2020. **a** Direct personal GHG emissions; **b** Indirect personal GHG emissions. (CE reflected the GHG emission factor effect, GI represented the energy structure effect, TS reflected the energy price effect, W represented the energy consumption effect; ED reflected the GHG emission intensity effect; NU was the consumption structure effect. WT reflected the consumption willing effect. GT represented the income level effect

The driving forces and their contribution to the variations of the Shanghainese P_GHG_ emissions from 2010 to 2020 were studied based on the LMDI model, which was shown in Section 2.4. The detailed results can be seen in Fig. 7. For DP_GHG_ emissions, it could be found that energy consumption was the main factor contributing to the growth of the GHG emissions (296.4%), and the GHG emission factor effect, energy price effect and energy structure effect were the factors to suppress GHG emission growth, which accounted for around − 92.2%, − 89.6% and − 14.6%, respectively. 2013 and 2016 were the 2 years with the largest increase, and 2014 was the year with the largest decrease in DP_GHG_ emissions during the observation period, which was mainly attributed to more energy consumption driven by the unusual weather as shown in Fig. 7. It could also be found that with the improvement of the living level, the more expenditure was spent on the energy, which correspondingly created more GHG emissions. While the adjustment of the energy structure that phasing out the traditional high-emission fuels (such as coal, gas and LPG), and using more clean energy could reduce GHG emissions.

The IP_GHG_ emissions decreased from 2010 to 2020 generally. The GHG intensity effect was the largest contributor to the reduction, with the value of around − 188.1%, followed by the consumption willingness (− 69.2%) and consumption structure (− 12.0%). The income level was the primary factor in inducing GHG emissions growth (169.3%). The decrease in the GHG intensity of consumption sectors was the main factor for the decrease in IP_GHG_ emissions from 2011 to 2013. The increase of the income level was the driving force for the quick increase of IP_GHG_ emissions from 2014 to 2018. It could also be found that the decline in the consumption willingness due to the COVID-19 lockdown measures was the main reason for the decline in IP_GHG_ emissions in 2020.

### The impacts of the methodology on the results

The previous studies on personal or household GHG emissions are shown in the SI. It could be found that the P_GHG_ emissions were divided into direct emissions and indirect emissions, while the boundary was from the resource obtaining to the product using in most previous studies. As was shown in Fig. 1, the generation and disposal of waste were involved in the study. And the boundary was extended from resource obtaining to the waste disposal. The results indicated that the emissions from the waste disposal accounted for around 3.1% of the total P_GHG_ emissions from 2010 to 2018, which were neglected in most studies. Affected by the forced source separation policy, the emissions decreased sharply and only accounted for around 0.4% of the total emissions in 2020. It was also foreseeable that waste disposal could even be a negative carbon source for residents with the improvement of the waste source separation and recycling habits. The waste generation and disposal seemed to be a potential source to reduce P_GHG_ emissions and could not be neglected [2].

### The comparison of Shanghainese P_GHG_ emissions with others

**Fig. 8:**
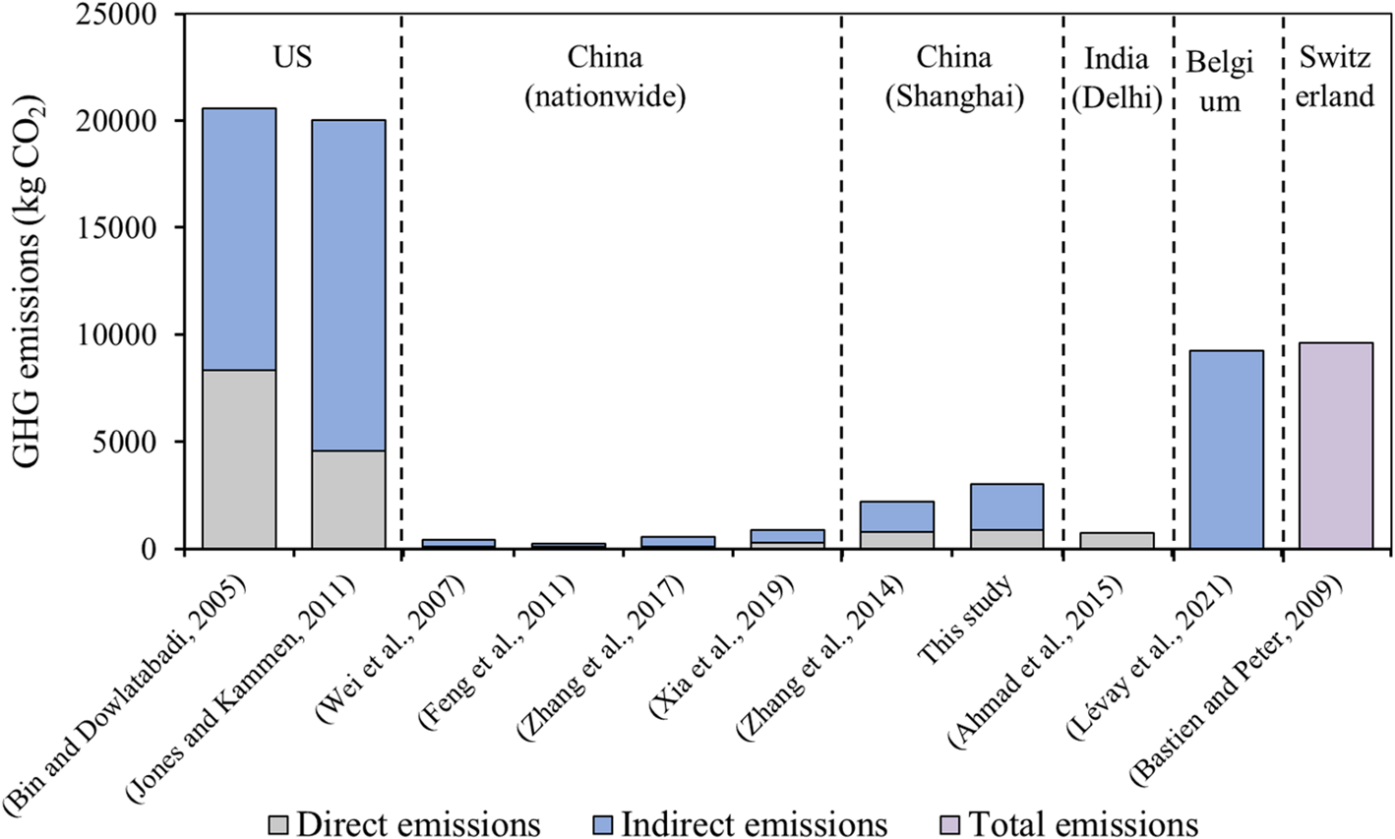
Summary of P_GHG_ emissions in different areas

The P_GHG_ emissions from different sectors were compared with the previous studies in different areas and the results are shown in Fig. 8. It was found that the American had the highest P_GHG_ emissions, followed by the Europeans [4, 16], and the Chinese P_GHG_ emissions were similar with the Indian. Shanghai, China’s most economically developed city, the average P_GHG_ emissions were around 3 to 4 times of the national average value in 2010. However, the American average P_GHG_ emissions were around 6.6 times of the Shanghainese P_GHG_ emissions.

Comparing the DP_GHG_ emissions in Shanghai with Delhi, the second largest city in India, although the energy consumption was higher in Shanghai than in Delhi, the DP_GHG_ emissions were similar [1], which may be attributed to the lower energy emission factor in China (India had a higher share of traditional biomass and kerosene and lower access to electricity than China) [26]. For indirect GHG emissions from personal consumption, food was the largest expenditure and kept increasing in recent years, which also contributed to the largest GHG emissions in Shanghai. The results were consistent with the report by [11]. However, it should be noted that the emissions from education, cultural and recreation services occupied the second largest source of the IP_GHG_ emissions, and even exceeded that from food in some years in Shanghai from 2010 to 2020, which was much higher than the previous studies [11, 39]. It could also reflect that the residents in Shanghai, where as one of the most developed cities in China, paid more attention on education and entertainment.

### The policy implications for P_GHG_ emissions reductions

It could be found that the reduction of P_GHG_ emissions was not only realized by relying on personal low-carbon behaviors. Considering the DP_GHG_ emissions, except for reducing the daily energy consumption, the GHG emission factor and the energy structure were crucial points for reducing emissions. While it should be noted that the energy consumption was hard to reduce in order to maintain our life quality. Under this circumstance, the revolution of the energy system to reduce the energy GHG emission factors was a feasible method, and one of the key methods was to change the use of fossil fuels into clean energy.

As for IP_GHG_ emissions, it could be found that the reduction of GHG intensity of consumptions was the most important contributor to IP_GHG_ emission reductions, and we could also conclude that the purchase of low GHG intensity products could help realize low P_GHG_ emissions lifestyles without lowering our living standards. The enterprises should increase the proportion of clean energy and improve the energy efficiency in production to promote low-carbon economy, which was conducive to the reduction of our P_GHG_ emission.

When making policies, China has faced the dilemma that it was necessary to encourage personal consumptions to ensure domestic economic growth, while also need to mitigate the rapid increase of GHG emissions to respond to the international pressure of decreasing GHG emissions. Multi-dimension measures should be taken to reduce P_GHG_ emissions. For the Chinese government, some low carbon policies such as the carbon labelling and green levy programs should be announced in which the higher GHG intensity commodities should be charged with the higher levies, which could encourage the industries to develop low carbon technologies and equipment. Meanwhile, the energy structure should be optimized, of which the clean energy could take more proportion, reducing the emission factors of the electricity. For individuals, the low carbon lifestyle should be developed. The low GHG intensity products and services were encouraged to be consumed, which would also force the companies to make low-carbon products. Besides, the resource and materials should be saved and reused, the waste source separation and recycling should be encouraged.

Finally, limitations also existed in our study. The study was based on the previous year books, while single data points without standard deviations were available, and that is why there were no error bars in the figures. Besides, limited by the available data, a premise of the CLA method was that all products within an industry had the same GHG intensity, which may emerge some errors for individuals when applying the model accounting the personal GHG emissions. More detailed product categories and the corresponding GHG intensity could be provided in the subsequent studies.

## Conclusions

This study put forward the boundary and methodology for the quantifying of GHG emissions at the personal level, which extended from resource obtaining to waste disposal. The Shanghainese average P_GHG_ emissions from 2010 to 2020 were assessed as a case study. The results showed that the P_GHG_ emissions decreased from 3796.05 kg CO_2_ (2010) to 3046.87 kg CO_2_ (2014) and then increased to 3411.35 kgCO_2_ (2018), with a rise after a decline changing trend in the stable development period. The IP_GHG_ emissions took the most, with the proportion of around 65.2% of the total emissions. The P_GHG_ emissions decreased by around 0.53 kg CO_2_ (0.02%) and 405.86 kg CO_2_ (12.9%) in 2019 and 2020, which was mainly affected by the waste forced source separation policy and the COVID-19 pandemic. The P_GHG_ emissions from waste disposal were found to be around 3.1% of the total emissions, which was neglected in most previous studies. The LMDI model was developed to decompose the driving forces of the P_GHG_ emissions, and it was found that energy consumption effect was the main factor contributing to the growth of the DP_GHG_ emissions, and the GHG emission factor effect, energy price effect and energy structure effect were the factors to suppress DP_GHG_ emissions. The income level was the primary factor in inducing GHG emissions growth, and the consumption GHG intensity effect was the largest contributor to the reduction, followed by the consumption willingness and consumption structure. Finally, the Shanghainese P_GHG_ emissions were compared with other areas, and the policy implications such as carbon labelling, green levy programs, energy structure optimization and low carbon lifestyles of individuals were encouraged to reduce the P_GHG_ emissions.

## Supplementary Information

**Additional file 1: Table S1.** 100-year Global Warming Potential (GWP). **Table S2.** Emission factors for different MSW treatment technologies. **Table S3.** Overview of the studies on household or personal GHG emissions. **Table S4.** The household energy consumption per capita in Shanghai from 2010 to 2020. **Table S5.** The GHG intensity of different sectors from 2010 to 2020 (unit: t CO_2_/10^4^ yuan). **Table S6.** The waste amount and treatment methods in Shanghai from 2010 to 2020 (unit: ten thousand tons). **Table S7.** The total population of Shanghai from 2010 to 2020 (ten thousand people). **Table S8.** The emission factors of the different fuel types. **Table S9.** The electricity emission factor from 2010 to 2020 (kg CO_2_/kWh).

## Data Availability

The data and material and available in the supplementary information.

## Abbreviations

GHG: Greenhouse gas
P_GHG_ emissions: Personal GHG emissions
CO_2_: Carbon dioxides
DP_GHG_ emissions: Direct personal GHG emissions
IP_GHG_ emissions: Indirect personal GHG emissions
IP_GHG-C_ emissions: Indirect personal GHG emissions from consumptions
IP_GHG-W_ emissions: Indirect personal GHG emissions from waste disposal
SI: Supplementary information
LMDI: Logarithmic Mean Divisia Index
LCA: Life cycle assessment
LPG: liquefied petroleum gas
ECM: Emissions coefficient method
IPCC: Intergovernmental Panel on Climate Change
kg ce: kg of standard coal equivalent
CLA: Consumer lifestyle approach
CNY: Chinese Yuan
GWP: global warming potential
