# Supplementary material for: Personal GHG emissions accounting and the driving forces decomposition in the past 10 years
Source: Carb Neutrality. 2023 Feb 8;2(1):3. doi: 10.1007/s43979-023-00045-9 (PMC9905011; doi:10.1007/s43979-023-00045-9)
Supplement: Supplementary file 1 — Additional file 1: Table S1. 100-year Global Warming Potential (GWP). Table S2. Emission factors for different MSW treatment technologies. Table S3. Overview of the studies on household or personal GHG emissions. Table S4. The household energy consumption per capita in Shanghai from 2010 to 2020. Table S5. The GHG intensity of different sectors from 2010 to 2020 (unit: t CO2/104 yuan). Table S6. The waste amount and treatment methods in Shanghai from 2010 to 2020 (unit: ten thousand tons). Table S7. The total population of Shanghai from 2010 to 2020 (ten thousand people). Table S8. The emission factors of the different fuel types. Table S9. The electricity emission factor from 2010 to 2020 (kg CO2/kWh). [file 43979_2023_45_MOESM1_ESM.docx]

**Supplementary Information for:**

**Personal GHG emissions accounting and the driving forces decomposition in the past 10 years**

Yuxiao Zhou^1^, Jiyang Li^2^, Jicui Cui^1^, Hui Wang^1^, Chuan Wang^3^, Ruina Zhang^3^, Ying Zhu^4^, Nanwen Zhu^1^, Ziyang Lou^1,2,5,6*^

1 Shanghai Engineering Research Center of Solid Waste Treatment and Resource Recovery, School of Environmental Science and Engineering, Shanghai Jiao Tong University, Shanghai, 200240, China

2 China-UK Low Carbon College, Shanghai Jiao Tong University, Shanghai, 201306, China

3 Shanghai Environmental Sanitary Engineering Design Institute Co., Ltd, Shanghai, 200232, China

4 Advanced Materials Institute, Qilu University of Technology (Shandong Academy of Sciences), Jinan 250014, China

5 China Institute for Urban Governance, Shanghai Jiao Tong University, Shanghai, 200240, China

6 Shanghai Jiao Tong University Sichuan Research Institute

*Corresponding author: Ziyang Lou, Dr

Email: louworld12@sjtu.edu.cn

Address: 800 Dongchuan Road, Minhang District, Shanghai, China

Tel.: +8613564381973

Table S1 100-year Global Warming Potential (GWP)

| Gas | GWP（100-year) |
| --- | --- |
| CO_2_ | 1 |
| CH_4_ | 28 |
| N_2_O | 273 |

Table S2 Emission factors for different MSW treatment technologies

| Technologies | Emission factor | unit | source |
| --- | --- | --- | --- |
| Landfill | 552.07 | kg CO_2_-eq/t | (Cai et al., 2018; Liu et al., 2021; Yang et al., 2013) |
| Simple landfill | 819.5 | kg CO_2_-eq/t | (Yang et al., 2013) |
| Incineration | 110.3 | kg CO_2_-eq/t | (Liu et al., 2021; Lou et al., 2015; Yang et al., 2012) |
| Composting | 62.4 | kg CO_2_-eq/t | (Zhou et al., 2022) |
| Anaerobic digestion | -36.5 | kg CO_2_-eq/t | (Zhou et al., 2022) |
| Recycling | -428.5 | kg CO_2_-eq/t | (CPCD, 2022; Aren et al., 2022) |

Table. S3 Overview of the studies on household or personal GHG emissions

| **Study area** | **Year** | **Description** | **Methodology** | **Conclusion** | **Source** |
| --- | --- | --- | --- | --- | --- |
| United States | 1997 | Direct GHG emissions from energy consumption and indirect emissions from the preparation of products and services. | ECM  CLA | Indirect influences involve more than twice the direct energy use and CO_2_ emissions. | (Bin and Dowlatabadi, 2005) |
| China | 2007 | Direct GHG emissions from energy consumption and indirect emissions from the production of products and services. | ECM  CLA | For urban residents the indirect impact on energy consumption is 2.44 times greater than the direct impact. | (Wei et al., 2007) |
| China | 1999-2007 | Direct GHG emissions from energy consumption and indirect emissions from the preparation of products and services. | ECM  CLA | Indirect emissions were larger than direct emissions, while the gap was narrowing. | (Feng et al., 2011) |
| United States | 2005 | Emissions embodied in transportation, energy, water, waste, food, goods, and services. | LCA  IOM | The P_GHG_ was around 20 t, for the baseline of 2005. | (Jones and Kammen, 2011) |
| China | 2000-2010 | Direct emissions were related to direct household fuel use, and indirect emissions, are those that from the production and distribution processes of goods and services. | IOM | The indirect CO_2_ emission appear the main parts of total CO_2_ emission, accounting for 77–84%. | (Zhang et al., 2017) |
| Belgium | 2014 | Indirect emissions embedded in the supply chain of goods and services that consumed | IOM | GHG emissions grow from 6.1 to 12.4 t from the lowest to the highest income decile, and the income was the most important determinant. | (Lévay et al., 2021) |
| China | 1995-2009 | Direct emissions were related to direct household fuel use, and indirect emissions, are those that from the production and distribution processes of goods and services. | ECM  IOM | Urban employment, rapid urbanization, and the two-child policy have a magnifying effect on increasing indirect carbon emissions | (Xia et al., 2019) |
| Delhi | 2010 | Household emissions of direct energy use (electricity, cooking fuels, and private transportation) | ECM | Emissions from India’s cities are similar in magnitude to China’s cities but typically much lower than those of comparable U.S. cities | (Ahmad et al., 2015) |
| Shanghai | 2010 | Direct emissions were related to direct household fuel use, and indirect emissions, are those that from the production and distribution processes of goods and services. | Urban-RAM | Direct carbon emissions and indirect carbon emissions accounted for 64.1% and 35.9% | (Zhang et al., 2014) |
| Switzerland | 2009 | Emissions embodied in household consumption | LCA | The range 5–17 tons of CO_2_ per capita was found, which stemmed mainly from heating, electricity use, car use, and travel by aircraft | (Bastien and Peter, 2009) |

IOM—input output model; CLA—Consumer lifestyle approach

LCA—Life cycle assessment; ECM—Emission coefficient method

Table S4 The household energy consumption per capita in Shanghai from 2010 to 2020

|  | 2010 | 2011 | 2012 | 2013 | 2014 | 2015 | 2016 | 2017 | 2018 | 2019 | 2020 |
| --- | --- | --- | --- | --- | --- | --- | --- | --- | --- | --- | --- |
| Coal (kg) | 20.75 | 18.64 | 17.42 | 14.21 | 13.87 | 12.48 | 7.95 | 1.62 | 1.62 | 1.65 | 1.61 |
| Natural gas (m^3^) | 34.52 | 37.12 | 42.01 | 46.08 | 48.46 | 55.77 | 58.95 | 58.86 | 64.07 | 65.08 | 68.94 |
| LPG (kg) | 14.65 | 14.71 | 14.92 | 16.28 | 14.49 | 10.42 | 9.08 | 7.10 | 7.08 | 7.06 | 6.34 |
| Electricity (kWh) | 748.74 | 753.61 | 792.66 | 855.12 | 718.43 | 766.34 | 900.60 | 927.47 | 985.70 | 988.71 | 1034.83 |
| Coal gas (m^3^) | 27.88 | 23.14 | 16.54 | 11.14 | 4.83 | 0 | 0 | 0 | 0 | 0 | 0 |
| Gasoline (kg) | 51.78 | 60.70 | 70.94 | 77.93 | 85.33 | 97.61 | 105.52 | 116.76 | 89.00 | 89.99 | 84.60 |
| Diesel (kg) | 18.19 | 17.91 | 17.89 | 19.66 | 17.62 | 17.69 | 17.85 | 14.01 | 3.92 | 4.12 | 3.62 |

Table S5 The GHG intensity of different sectors from 2010 to 2020 (unit: t CO_2_/10^4^ yuan)

|  | 2010 | 2011 | 2012 | 2013 | 2014 | 2015 | 2016 | 2017 | 2018 | 2019 | 2020 |
| --- | --- | --- | --- | --- | --- | --- | --- | --- | --- | --- | --- |
| Food | 1.13 | 0.90 | 0.77 | 0.70 | 0.70 | 0.74 | 0.73 | 0.70 | 0.71 | 0.68 | 0.67 |
| Clothing | 1.72 | 1.53 | 1.48 | 1.42 | 1.40 | 1.25 | 1.11 | 1.17 | 1.11 | 1.08 | 1.06 |
| Household facilities, articles and services | 1.41 | 1.33 | 1.29 | 1.23 | 1.16 | 1.17 | 1.22 | 1.07 | 1.05 | 1.07 | 1.01 |
| Communication services | 0.86 | 0.73 | 0.65 | 0.57 | 0.51 | 0.46 | 0.42 | 0.36 | 0.32 | 0.30 | 0.27 |
| Education, cultural and recreation services | 3.35 | 2.70 | 1.90 | 1.62 | 1.57 | 1.48 | 1.44 | 1.31 | 1.30 | 1.28 | 1.21 |
| Medicine and medical services | 0.80 | 0.71 | 0.60 | 0.52 | 0.47 | 0.42 | 0.38 | 0.34 | 0.30 | 0.29 | 0.25 |
| Residence | 1.39 | 1.34 | 1.31 | 1.18 | 1.17 | 1.21 | 1.09 | 1.12 | 1.11 | 1.09 | 1.06 |

Table S6 The waste amount and treatment methods in Shanghai from 2010 to 2020 (unit: ten thousand tons)

|  | 2010 | 2011 | 2012 | 2013 | 2014 | 2015 | 2016 | 2017 | 2018 | 2019 | 2020 |
| --- | --- | --- | --- | --- | --- | --- | --- | --- | --- | --- | --- |
| Incineration | 108 | 104 | 104 | 166 | 238 | 250 | 311 | 361 | 386 | 407 | 682 |
| Landfill | 418 | 394 | 379 | 378 | 329 | 329 | 325 | 358 | 387 | 324 | 70 |
| Simple Landfill | 83 | 63 | 36 | 2 | 0 | 0 | 0 | 0 | 0 | 0 | 0 |
| Food waste disposal | 90 | 114 | 145 | 155 | 129 | 143 | 116 | 128 | 144 | 192 | 115 |
| Recycle | 5 | 5 | 4 | 11 | 9 | 15 | 17 | 21 | 47 | 101 | 210 |
| Others | 27 | 25 | 47 | 25 | 37 | 42 | 111 | 32 | 19 | 14 | 8 |
| Total | 732 | 704 | 716 | 736 | 743 | 779 | 880 | 900 | 984 | 1038 | 1085 |

Table S7 The total population of Shanghai from 2010 to 2020 (ten thousand people)

|  | 2010 | 2011 | 2012 | 2013 | 2014 | 2015 | 2016 | 2017 | 2018 | 2019 | 2020 |
| --- | --- | --- | --- | --- | --- | --- | --- | --- | --- | --- | --- |
| Population | 2302.66 | 2347.46 | 2380.43 | 2415.15 | 2425.68 | 2415.27 | 2419.7 | 2418.33 | 2423.78 | 2428.14 | 2488.20 |

Table S8 The emission factors of the different fuel types

|  | Coal（kg） | Natural gas (m^3^) | LPG (kg) | Coal gas（m^3^） | Gasoline (kg) | Diesel (kg) |
| --- | --- | --- | --- | --- | --- | --- |
| Emission factor | 2.03 | 2.80 | 5.11 | 1.45 | 3.85 | 3.82 |
| units | kg CO_2_/ kg | kg CO_2_/ m^3^ | kg CO_2_/ kg | kg CO_2_/ m^3^ | kg CO_2_/ kg | kg CO_2_/ kg |

The emission factors were the life cycle emissions, including the emissions in supply chain, which came from the "China Products Carbon Footprint Factors Database" (CPCD, 2022)

Table S9 The electricity emission factor from 2010 to 2020 (kg CO_2_/kWh)

|  | 2010 | 2011 | 2012 | 2013 | 2014 | 2015 | 2016 | 2017 | 2018 | 2019 | 2020 |
| --- | --- | --- | --- | --- | --- | --- | --- | --- | --- | --- | --- |
| Emission factor | 0.68 | 0.69 | 0.67 | 0.66 | 0.65 | 0.63 | 0.62 | 0.62 | 0.62 | 0.61 | 0.58 |

**Reference:**

Ahmad, S., Baiocchi, G., Creutzig. 2015. CO2 emissions from direct energy use of urban households in India. Environmental Science & Technology, 49 (19), 11312-1132.

Aren, U., Ardolino, F. 2022. Technical and environmental performances of alternative treatments for challenging plastics waste. Resources Conservation & Recycling, 183, 106379.

Bastien, G., Peterde, D. H., 2009. GHG reduction potential of changes in consumption patterns and higher quality levels: Evidence from Swiss household consumption survey. Energy Policy, 37(12), 5650-5661.

Bin, S., Dowlatabadi, H. 2005. Consumer lifestyle approach to US energy use and the related CO2 emissions. Energy Policy, 33 (2), 197-208.

China City Greenhouse Gas Working Group (CPCD), 2022. China Products Carbon Footprint Factors Database. Beijing.

Jones, C. M., Kammen, D. M. 2011. Quantifying carbon footprint reduction opportunities for US households and communities. Environmental Science & Technology, 45(9), 4088–4095.

Lévay, P.Z., Vanhille, J., Goedemé, T., et al. 2021. The association between the carbon footprint and the socio-economic characteristics of Belgian households. Ecological economics, 186, 107065.

Wei, Y., Liu, L., Fan, Y., et al. 2007. The impact of lifestyle on energy use and CO_2_ emission: An empirical analysis of China’s residents. Energy Policy, 35, 247–257.

Yang, N., Zhang, Hua., Chen, Miao., et al. 2012. Greenhouse gas emissions from MSW incineration in China: Impacts of waste characteristics and energy recovery. Waste Management, 32, 2552-2560.

Yang, N., Zhang, Hua., Shao, Li-Ming., et al. 2013. Greenhouse gas emissions during MSW landfilling in China: Influence of waste characteristics and LFG treatment measures. Journal of Environmental Management, 129, 510-521.

Zhang, Y., Bian, X., Tan, W., et al. 2017. The indirect energy consumption and CO2 emission caused by household consumption in China: An analysis based on the input-output method. Journal of Cleaner Production, 163(1), 69–83.

Zhou, Y., Hu, Y., Chen, A.J.Y., et al. 2022. Environmental impacts and nutrient distribution routes for food waste separated disposal on large-scale anaerobic digestion/ composting plants. Journal of Environmental Management, 318 (15), 115624.
